# Decoupling Control of Micromachined Spinning-Rotor Gyroscope with Electrostatic Suspension

**DOI:** 10.3390/s16101747

**Published:** 2016-10-20

**Authors:** Boqian Sun, Shunyue Wang, Haixia Li, Xiaoxia He

**Affiliations:** Department of Precision Instrument, Tsinghua University, Beijing 100084, China; sbq12@mails.tsinghua.edu.cn (B.S.); wsy15@mails.tsinghua.edu.cn (S.W.); li-hx03@mails.tsinghua.edu.cn (H.L.)

**Keywords:** decoupling control, gyroscope rebalance loop, stiffness compensation, inner-loop decoupling compensator, outer-loop decoupling compensator, micromachined spinning-rotor gyroscope, electrostatic suspension

## Abstract

A micromachined gyroscope in which a high-speed spinning rotor is suspended electrostatically in a vacuum cavity usually functions as a dual-axis angular rate sensor. An inherent coupling error between the two sensing axes exists owing to the angular motion of the spinning rotor being controlled by a torque-rebalance loop. In this paper, a decoupling compensation method is proposed and investigated experimentally based on an electrostatically suspended micromachined gyroscope. In order to eliminate the negative spring effect inherent in the gyroscope dynamics, a stiffness compensation scheme was utilized in design of the decoupled rebalance loop to ensure loop stability and increase suspension stiffness. The experimental results show an overall stiffness increase of 30.3% after compensation. A decoupling method comprised of inner- and outer-loop decoupling compensators is proposed to minimize the cross-axis coupling error. The inner-loop decoupling compensator aims to attenuate the angular position coupling. The experimental frequency response shows a position coupling attenuation by 14.36 dB at 1 Hz. Moreover, the cross-axis coupling between the two angular rate output signals can be attenuated theoretically from −56.2 dB down to −102 dB by further appending the outer-loop decoupling compensator. The proposed dual-loop decoupling compensation algorithm could be applied to other dual-axis spinning-rotor gyroscopes with various suspension solutions.

## 1. Introduction

Miniature spinning-rotor gyroscopes have attracted much attention in recent decades and have become a promising technology to offer tactical and inertial grade performances [1,2]. The traditional spinning-rotor gyroscopes [3], as the most highly-accurate gyroscopes in inertial navigation and pointing applications, function as a two-degree-of-freedom angular rate or position sensor with various suspension techniques [3,4,5]. In order to minimize the mechanical friction of the spinning rotor and achieve high accuracy, different miniaturized gyroscope suspension mechanisms, such as contactless electrostatic and electromagnetic bearings [6,7,8] and liquid-suspended and gas-lubricated bearings [9,10,11], have been studied and developed. Among them, electrostatic suspension is comparatively compatible with existing microfabrication techniques and ideally suited for micromachined spinning-rotor gyroscopes. Moreover, the small mass of the rotor (~10^−6^ kg) allows for low suspension voltage and makes it possible for the MEMS device to integrate the gyro electronics into the same package.

The spinning rotor of the micromachined electrostatically suspended gyroscope (MESG) is suspended in a vacuum cavity by a contactless electric bearing with five degrees of freedom (DOFs), which eliminates the friction and wear inherent in mechanical bearings. It is one of the most promising candidates offering high performance, low cost, and miniature multi-axis inertial sensors [12,13]. Currently, several MESG prototypes were reported to offer tactical-grade level performance [14] and aim to achieve navigation grade performance [15]. Tokimec Inc. (Japan) has reported several prototypes of MESGs [14,16]. Auburn University (USA) found that the noise levels of such sensor were improved after pre- and post-radiation tests [15]. Recently, the University of Southampton (UK) presented the design and fabrication of a MESG with triple-wafer stack bonding [17]. Shanghai Jiaotong University (China) reported the hybrid microfabrication and successful 5-DOF suspension of a MESG [18].

The development of such a high-precision micromachined spinning-rotor gyroscope requires a series of challenging techniques, such as microfabrication of the glass/silicon/glass triple structure, electrostatic suspension of the rotor in five DOFs, spin-up of the rotor over 10^4^ r/min in vacuum, and rotor spinning with constant speed for angular momentum conservation [19]. Based on our prior work, such as the fabrication process by bulk micromachining [20], designing and testing an active electrostatic bearing [7], and performance testing of the MESG in vacuum [21], the micromachined device can be used as a dual axis angular rate gyroscope by spinning-up the rotor rate over 10^4^ r/min [19]. Since the MESG is a kind of free spinning rotor gyroscope, an inherent coupling error resulting from a rigid body rotation controlled by a torque-rebalance loop exists in this dual-axis angular rate sensor. Similar to the dynamically tuned gyroscope [22], the inherent cross-axis coupling error between the dual-axis torque-rebalance loop greatly limits the potential accuracy improvement of MESGs. Therefore, it is necessary to design and realize decoupling control of the dual-axis angular rate gyroscope that aims to achieve tactical-grade level or better performance. Currently, most of the reported results focus on microfabrication and device optimization [17,20,23], capacitive position sensing [24,25,26] and suspension control [18,27,28], rotor spin-up [21,28,29], design of the gyro rebalance loop [21,30] and error analysis of the gyro [31]. Little work has been reported on the cross-axis coupling effect, nor the decoupling control of the micromachined spinning rotor gyroscopes with electrostatic suspension.

This paper proposes and demonstrates experimentally a stiffness compensation method with an aim to eliminate the negative spring effect inherent in the spinning-rotor gyroscope dynamics and also increase active suspension stiffness. Further, for the dual-axis gyroscope with the proposed stiffness compensation, a dual-loop decoupling compensation is designed and investigated to attenuate the angular position and rate coupling error by considering the residual air-film damping effect. Finally, an electrical measurement method is proposed to provide an effective approach to validate experimentally the decoupling compensation in an MESG setup.

## 2. Design of Rebalance Loops

### 2.1. Description of the MEMS Spinning-Rotor Gyro

**Figure 1:**
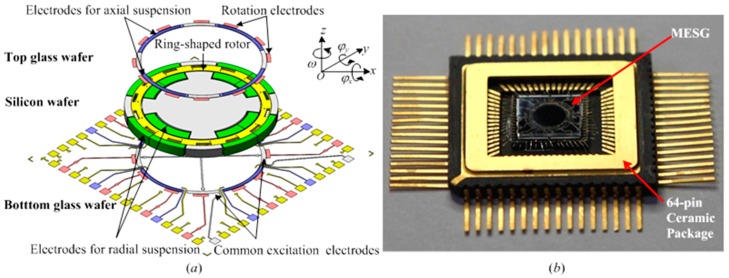
Micromachined dual-axis spinning-rotor gyroscopes (MESG): (**a**) the exploded view of the device and (**b**) a fabricated device.

The MESG device, which is a glass/silicon/glass triple stack structure and fabricated by bulk microfabrication, is primarily composed of a spinning rotor and associated stators. An exploded view of the MESG is depicted in Figure 1a, and a fabricated device with a 64-pin ceramic package is illustrated in Figure 1b.

The essential geometry of the MESG comprises a spinning ring-shaped rotor surrounded by sets of sensing, suspension and rotation electrodes [32]. The ring-shaped rotor has an outer radius 2.0 mm, an inner radius 1.73 mm and a thickness 68 μm. At a vacuum pressure below 0.2 Pa, the rotor is suspended and centered in an evacuated vacuum cavity by a contactless electrostatic bearing. It is driven by a three-phase electrostatic motor at a high speed over 10^4^ r/min. Moreover, a constant speed control loop is used to conserve the angular momentum of the spinning rotor [19]. The torque rebalance loop, consisting of the angular position pick-offs, loop controller, and electrostatic torquers, will output the measured dual-axis angular rate signals, in other words, it will measure around two orthogonal input axes, $\varphi_{x},\varphi_{y}$. The simulation results based on the parameters in Table 1 show the scale factor and measuring range of the MESG are 16.05 mV/°/s and ±196.1 °/s, respectively.

### 2.2. Stiffness Compensated Model

When the MESG operates at the angular rate measurement mode where the rotor is spinning at high speed, the linearized equations (extended from [21]) governing the gyro dynamics can be expressed as:

(1) $$\left\{ \begin{array}{l} J_{e}{\ddot{\theta }}_{x}+C{\dot{\theta }}_{x}+H{\dot{\theta }}_{y}+\lambda \theta_{y}=T_{x}-J_{e}{\ddot{\varphi }}_{x}-H{\dot{\varphi }}_{y}\\ J_{e}{\ddot{\theta }}_{y}+C{\dot{\theta }}_{y}-H{\dot{\theta }}_{x}-\lambda \theta_{x}=T_{y}-J_{e}{\ddot{\varphi }}_{y}+H{\dot{\varphi }}_{x} \end{array}\right.$$

where ${\dot{\varphi }}_{x},{\dot{\varphi }}_{y}$ denote the two external angular rate inputs with respect to inertial space. The angular position of the rotor with respect to the stator electrodes are $\theta_{x},\theta_{y}$. $J_{e},J_{z}$ represent the rotor moments of inertia about the *x*- and *z*- axes. $\omega_{z}$ is the rated speed of the rotor with respect to the gyro case, and $H=J_{z}\omega_{z}$ is the angular momentum of the rotor about the *z*-axis. C and λ=(C⋅ω) are the squeeze-film damping coefficient and the orthogonal-damping elastic coefficient about the *x*-, *y*-axes. $V_{x},V_{y}$ and $T_{x},T_{y}$ denote the control voltages produced by the rebalance loop and electrostatic torques applied on the rotor, respectively.

Ideally, the torquer accurately produces the electrostatic torque on the rotor, which is proportional to the control voltage and independent of the rotor angular position. However, considering the inherent negative stiffness effect, the electrostatic torquer can be modeled as:

(2) $$\left\{ \begin{array}{l} T_{x}=-K_{v}V_{x}+K_{n}\theta_{x}\\ T_{y}=-K_{v}V_{x}+K_{n}\theta_{y} \end{array}\right.$$

where $K_{v}$ and $K_{n}$ are the torquer gain and angular position stiffness of the electrostatic suspension loop. As the negative spring effect (from *K*_n_) on the electrostatic torque is dependent on the current rotor angular position, it is clear that the overall torque will be reduced for large rotor deviation. Hence, an electric compensator which aims to eliminate the effect of $K_{n}$ is proposed and expressed in Equation (3), where $K_{s}$ is the sensitivity of the angle position sensor and $K_{a}$ is the voltage amplifier gain. Then, the desirable torque characteristics, in other words, $T_{\mathrm{cx}}=-K_{v}V_{x}$, $T_{\mathrm{cy}}=-K_{v}V_{y}$ can be obtained by replacing the original control voltages (*V*_x_, *V*_y_) with $V_{\mathrm{cx}},V_{\mathrm{cy}}$. Here, $K_{c}$ is the compensator coefficient which is required to be calibrated by the experiment and leads to an accurate stiffness compensation by eliminating negative spring effect. The stiffness compensation is also beneficial in helping increase the electrostatic bearing stiffness and improve stability of the rebalance loop [4]. The main parameters of the rebalance loop are listed in Table 1. Simulation results show that a parameter mismatch, such as $J_{e}$ and C, will result in linearly increased coupling errors.

(3) $$\left\{ \begin{array}{l} V_{\mathrm{cx}}=V_{x}+K_{s}K_{a}K_{c}\theta_{x}\\ V_{\mathrm{cy}}=V_{y}+K_{s}K_{a}K_{c}\theta_{y}\\ K_{c}=K_{n}/(K_{s}K_{v}K_{a}) \end{array}\right.$$

### 2.3. Inner-Loop and Outer-Loop Decoupling Compensation

Similar to traditional dual-axis spinning-rotor gyroscopes such as the dynamically tuned gyro, the cross-axis coupling exists in the dual-axis torque rebalance loop of the MESG as well. Therefore, it is necessary to evaluate the inherent cross-coupling error resulting from the gyro rebalance loop and then find an effective decoupling method.

**Figure 2:**
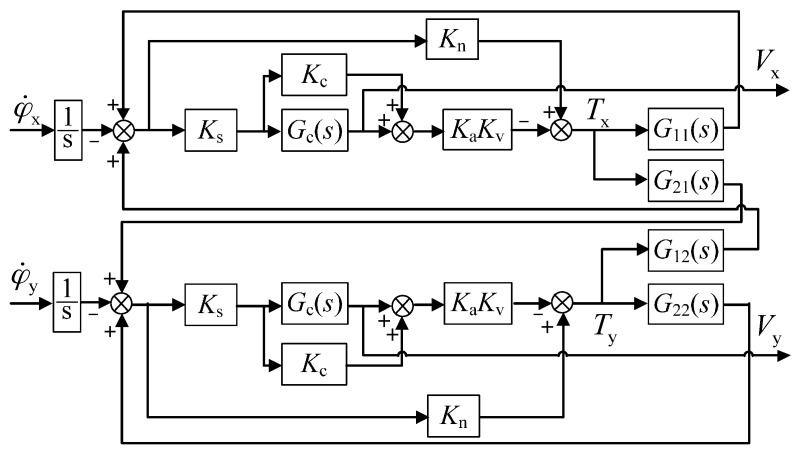
Block diagram of the rebalance loop without decoupling compensation.

Figure 2 shows the dual-axis rebalance loop before the decoupling compensation is applied. It is comprised of a pair of cross-axis loops and a pair of direct-axis loops. Equation (1) and $T_{\mathrm{cx}},T_{\mathrm{cy}}$ can be expressed in the form of a transfer function, using the Laplace transform, as:

(4) $$\left[\begin{array}{c} V_{x}\left(s\right)\\ V_{y}\left(s\right) \end{array}\right]=\frac{K_{s}G_{c}\left(s\right)}{I+K_{s}G_{c}\left(s\right)K_{a}K_{v}G\left(s\right)}\left(-\frac{1}{s}\right)\left[\begin{array}{c} {\dot{\varphi }}_{x}\left(s\right)\\ {\dot{\varphi }}_{y}\left(s\right) \end{array}\right]$$

where G(s) describes the dynamics of the spinning rotor in the form of

(5) $$\begin{array}{l} G(s)=\left[\begin{array}{ll} G_{11}(s) & G_{12}(s)\\ G_{21}(s) & G_{22}(s) \end{array}\right],\\ \{\begin{array}{c} G_{11}(s)=G_{22}(s)=(J_{e}s^{2}+Cs)/\Delta (s)\\ G_{21}(s)=-G_{12}(s)=(Hs+\lambda )/\Delta (s)\\ \Delta (s)={\left(J_{e}s^{2}+Cs\right)}^{2}+{\left(Hs+\lambda \right)}^{2} \end{array} \end{array}$$

Specifically, $G_{12}(s),G_{21}(s)$ and $G_{11}(s),G_{22}(s)$ indicate the direct-axis terms and coupling-axis terms in the loop, which describe the precession motion and rigid body characteristics of the spinning-rotor gyro, respectively.

Equation (4) describes the relationship between the rate input ${\dot{\varphi }}_{x},{\dot{\varphi }}_{y}$ and the gyro output $V_{x},V_{y}$ of the rebalance loop, in other words, the inputs and outputs of the dual-axis angular rate sensor. For instance, the cross-axis loop from external angular rate input ${\dot{\varphi }}_{x}$ to output voltage $V_{y}(s)$ in Figure 2 is the gyro sensing loop, considering that when an input ${\dot{\varphi }}_{x}$ occurs, the output $V_{y}(s)$ reflects the corresponding input change, while the direct-axis loop from ${\dot{\varphi }}_{x}$ to $V_{x}(s)$ is the coupling loop. On account of the damping coefficient C and λ, the unexpected coupling error from the rebalance loop model can be expressed by taking the Laplace transform

(6) $$\frac{V_{x}\left(s\right)/L[{\dot{\varphi }}_{x}]}{V_{y}\left(s\right)/L[{\dot{\varphi }}_{x}]}=\frac{V_{x}\left(s\right)}{V_{y}\left(s\right)}=\frac{\left(1+K_{s}K_{a}K_{v}G_{11}G_{c}\right)}{\left(K_{s}K_{a}K_{v}G_{12}G_{c}\right)}\approx \frac{\left(J_{e}s^{2}+Cs\right)}{\left(Hs+\lambda \right)}$$

Equation (6) shows that the cross-axis coupling is closely related to the damping coefficient and frequency of the angular rate input. The lower the squeeze-film damping and the frequency of input rate, the smaller the coupling error will be. Additionally, the vacuum condition for the operation of the spinning rotor and the rated spin rate $\omega_{z}$ will influence C and λ, respectively. Simulation results indicate that the inherent coupling is almost proportional to the frequency of rate input if the squeeze-film damping is limited to less than 10^−12^ Nm·s·rad^−1^, in other words, at vacuum pressure below 0.1 Pa.

**Figure 3:**
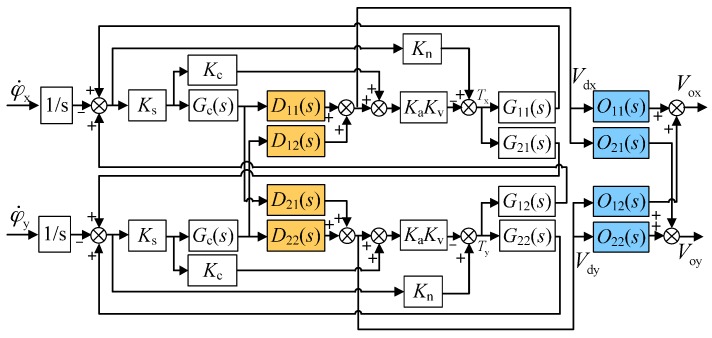
Block diagram of the rebalance loop with full decoupling compensation.

To suppress such coupling error, a pair of decoupling compensators, in other words, the inner-loop compensator D(s) and the outer-loop compensator O(s) as shown in Figure 3, is proposed and inserted into the rebalance loop. Firstly, the inner-loop decoupling compensator D(s) is introduced inside the rebalance loop considering that the residual air-film damping effect cannot be ignored. The proposed inner-loop compensator is

(7) $$D(s)=[\begin{array}{l} D_{11}(s)\\ D_{21}(s) \end{array}\begin{array}{l} D_{12}(s)\\ D_{22}(s) \end{array}]=\left[\begin{array}{cc} 1 & \frac{Hs+\lambda }{J_{e}s^{2}+Cs}\\ \frac{-(Hs+\lambda )}{J_{e}s^{2}+Cs} & 1 \end{array}\right]$$

The similar derivation about the relationship between the inputs ${\dot{\varphi }}_{x},{\dot{\varphi }}_{y}$ and the outputs $V_{\mathrm{dx}}(s),V_{\mathrm{dy}}(s)$ from the inner-loop compensators in Figure 3 can be expressed as:

(8) $$\left[\begin{array}{c} V_{\mathrm{dx}}\left(s\right)\\ V_{\mathrm{dy}}\left(s\right) \end{array}\right]=\frac{K_{s}G_{c}\left(s\right)DK_{a}}{I+K_{s}G_{c}\left(s\right)DK_{a}K_{v}G}\left(-\frac{1}{s}\right)\left[\begin{array}{c} {\dot{\varphi }}_{x}\left(s\right)\\ {\dot{\varphi }}_{y}\left(s\right) \end{array}\right]$$

It can be noticed from Equation (8) that the cross-axis coupling between the two output voltages $V_{\mathrm{dx}}(s),V_{\mathrm{dy}}(s)$ still exists after the inner-loop compensation is applied. In fact, the inner-loop compensator D(s) can effectively reduce the coupling between dual-axis angular positions ($\varphi_{x}$ and $\varphi_{y}$), yet needs to be combined with the outer-loop decoupling compensator O(s) as in Equation (9) so as to eliminate the coupling between two output voltages and achieve full decoupling. It should be noted that the design of O(s) is based on the inner-loop compensated system.

(9) $$O(s)=[\begin{array}{l} O_{11}(s)\\ O_{21}(s) \end{array}\begin{array}{l} O_{12}(s)\\ O_{22}(s) \end{array}]=\left[\begin{array}{cc} \frac{{\left(Hs+\lambda \right)}^{2}}{\Delta o(s)} & \frac{\left(J_{e}s^{2}+Cs)\;(Hs+\lambda \right)}{\Delta o(s)}\\ \frac{-(J_{e}s^{2}+Cs)\;(Hs+\lambda )}{\Delta o(s)} & \frac{{\left(Hs+\lambda \right)}^{2}}{\Delta o(s)} \end{array}\right]$$

where $\Delta o(s)={\left(J_{e}s^{2}+Cs\right)}^{2}+{\left(Hs+\lambda \right)}^{2}$.

Benefitting from the inner-loop and outer-loop compensators, the output voltages $V_{\mathrm{ox}}(s),V_{\mathrm{oy}}(s)$ of the two sensing axes separate ideally from each other, which is derived as in Equation (10).

(10) $$\left[\begin{array}{c} V_{\mathrm{ox}}\left(s\right)\\ V_{\mathrm{oy}}\left(s\right) \end{array}\right]=\frac{-K_{s}G_{c}\left(s\right)(Hs+\lambda )}{J_{e}s^{2}+Cs+K_{s}K_{a}G_{c}\left(s\right)K_{v}}\frac{1}{s}\left[\begin{array}{cc} 0 & 1\\ -1 & 0 \end{array}\right]\left[\begin{array}{c} {\dot{\varphi }}_{x}\left(s\right)\\ {\dot{\varphi }}_{y}\left(s\right) \end{array}\right]$$

Theoretically, by comparing Equations (4) and (10), the proposed decoupling compensation algorithm composing both the inner-loop and outer-loop compensators can eliminate completely the cross-axis coupling between dual-axis angular rate outputs.

**Figure 4:**
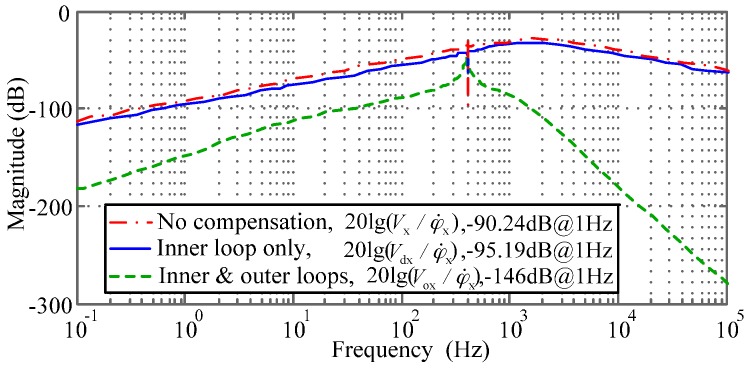
Comparison of the cross-axis frequency responses with and without decoupling compensation. The calculation of 20 lg($V_{x}/{\dot{\varphi }}_{x}$), 20 lg($V_{\mathrm{dx}}/{\dot{\varphi }}_{x}$), and 20 lg($V_{\mathrm{ox}}/{\dot{\varphi }}_{x}$) are based on Figure 2 and Figure 3.

Further, the different output voltages $V_{x}(s),V_{\mathrm{dx}}(s)$, and $V_{\mathrm{ox}}(s)$ as shown in Figure 4 can reflect the effect of the decoupling compensation when an angular rate input ${\dot{\varphi }}_{x}$ is applied. Benefitting from the accurate stiffness compensation, the frequency responses of the coupling error for different situations, including no decoupling compensation, inner-loop compensation only, and both the inner and outer-loop compensation, can be obtained by Equations (4), (8) and (10). Figure 4 illustrates the simulated coupling error responses via Simulink (Matlab). The inner-loop decoupling compensator D(s) does not effectively reduce the coupling of the angular rate response inside the rebalance loop. While it is combined with the outer-loop decoupling compensator O(s), the coupling error is reduced greatly, for example, up to a theoretical attenuation of 50.8 dB at 1 Hz. Moreover, the cross-axis coupling effect is observably attenuated by the decoupling compensation with increasing frequency of the angular rate input, which could result in a significant accuracy improvement on MESGs. On the other hand, it was found that the coupling effect caused by the gyro installation misalignment in turntable experiments generally reaches about −80 dB. Considering the coupling error produced by the rebalance loop can be reduced down to below −120 dB, which could be submerged in the large installation error response, the experimental validation of the decoupled control loop needs to be redesigned further.

### 2.4. Verification Scheme for the Inner and Outer Loop Decoupling

**Figure 5:**
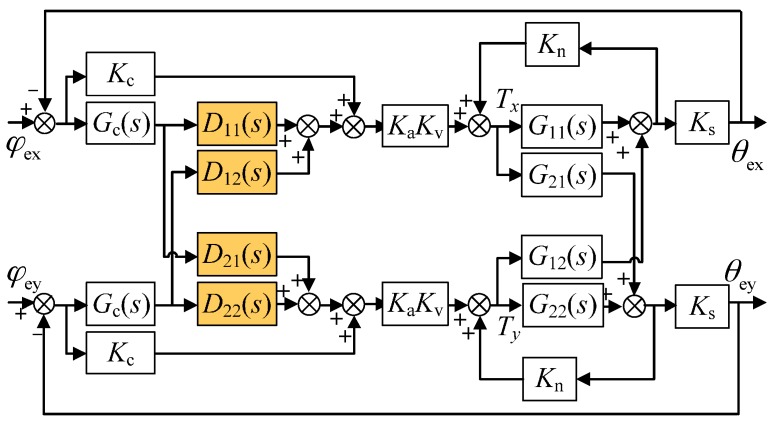
Block diagram of the rebalance loop in verification of the inner-loop decoupling compensation.

On account of the large installation misalignment effect and limited frequency range of the angular rate input during traditional turntable tests, we need to find a new solution to verify how the decoupling control works. Based on the decoupling compensator design in Section 2.3, this section introduces an indirect test scheme to verify the inner-loop and outer-loop compensator parameters step by step. A test scheme using an electric angular displacement signal as excitation, rather than the physical angular rate input, is proposed in this section and is aimed at proving the decoupling compensation method. Figure 5 illustrates schematically the resulted rebalance loop in test of the inner-loop decoupling compensation. An electric angular position ($\varphi_{\mathrm{ex}}(s)=K_{s}\cdot \varphi_{x}(s)$) is set as the input while the output $\theta_{\mathrm{ey}}(s)=K_{s}\cdot \theta_{y}(s)$ reflects the angular position output of the coupling-axis. If there is no inner-loop decoupling compensator, by removing D(s) in Figure 5, then the coupling of the two angular position responses remains and can be formulated as:

(11) $$\frac{\theta_{\mathrm{ey}}\left(s\right)}{\varphi_{\mathrm{ex}}\left(s\right)}=\frac{\frac{G_{c}(s)K_{a}K_{v}(-K_{s})G_{21}(s)}{{\left[1+G_{c}(s)K_{a}K_{v}K_{s}\cdot G_{11}(s)\right]}^{2}}}{1+{\left[\frac{G_{c}(s)K_{a}K_{v}K_{s}G_{12}(s)}{1+G_{c}(s)K_{a}K_{v}K_{s}\cdot G_{11}(s)}\right]}^{2}}$$

However, after the inner-loop decoupling compensator D(s) is introduced into the rebalance loop as shown in Figure 5, the output responses of the dual-axis angular position inside the loop are ideally decoupled,

(12) $$\theta_{\mathrm{ey}}\left(s\right)/\varphi_{\mathrm{ex}}\left(s\right)=0$$

**Figure 6:**
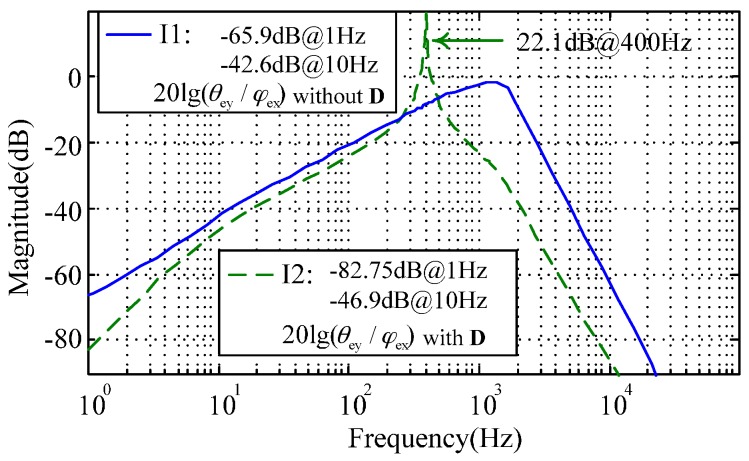
Simulated coupling error of the angular position that can be attenuated by the inner loop decoupling ***D***(s) (denoted by I2) compared with the rebalance loop without decoupling (I1).

Equation (12) indicates the inner-loop decoupling compensation can eliminate the cross-axis coupling between the two angular position responses. Simulation results illustrated in Figure 6 show that the inner-loop decoupling (denoted by I2) can attenuate the coupling response of the angular position by 16.85 dB at 1 Hz. Besides, the resonance peak at 400 Hz demonstrates the nutation frequency response of the MESG operating at about twice the rated spin rate (200 Hz), which benefits from the proper stiffness compensation.

**Figure 7:**
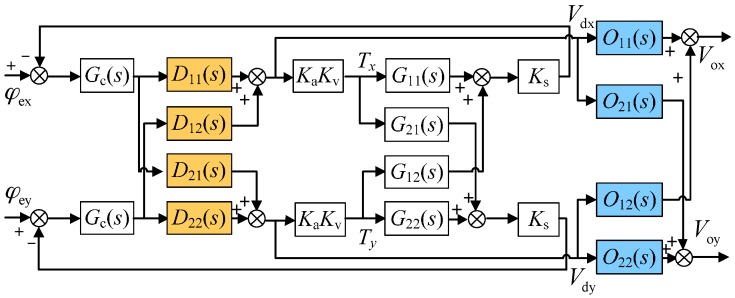
Block diagram of the rebalance loop in verification of the outer loop decoupling compensation.

**Figure 8:**
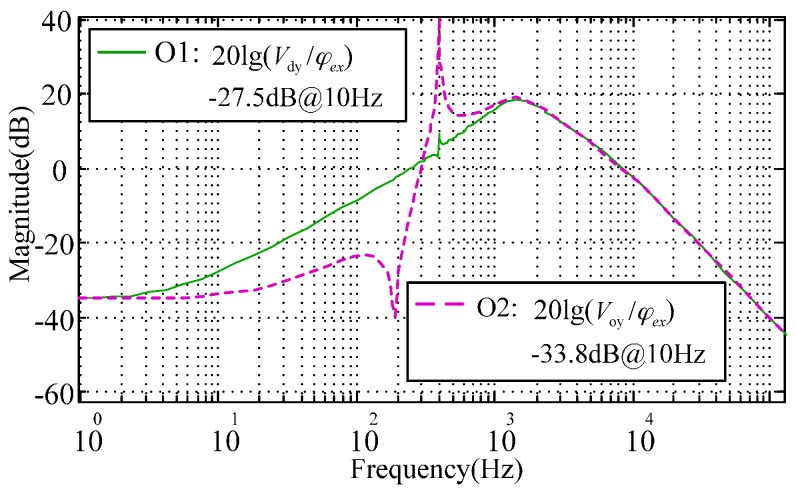
Simulated cross-axis coupling of the gyroscope output responses with (O2) and without (O1) the outer loop decoupling compensator.

Further, the full decoupling of the rebalance loop can be realized by appending the outer-loop decoupling compensator O(s). Figure 7 presents a test scheme to verify the characteristics of O(s) by setting $\varphi_{\mathrm{ex}}(s)$ as the input and $V_{\mathrm{dy}}(s),V_{\mathrm{oy}}(s)$ as the responses of the coupling-axis outputs, respectively. Figure 8 compares the simulated cross-axis coupling responses with and without the outer loop decoupling compensators, where the inner-loop and outer-loop decoupling curve, O2, and the inner-loop decoupling curve, O1, correspond to the loop transfer functions $V_{\mathrm{oy}}(s)/\varphi_{ex}(s)$ and $V_{\mathrm{dy}}(s)/\varphi_{ex}(s)$, respectively. Comparing Figure 8 with Figure 4, it is noted that the cross-axis coupling error becomes much higher. This is because the angular position usually keeps zero for the MESG operating at the angular rate measurement mode, and the input of the gyro rebalance loop is the external rate as shown in Figure 4. However, the input of Figure 8 is an electric angular position which is only used in the proposed electrical measurement mode to validate the decoupling compensation. It is clearly shown that the outer-loop compensation can suppress the coupling-axis output effectively in a frequency range of less than 200 Hz, for example, an attenuation by 6.3 dB at 10 Hz. The resonance peak of the nutation frequency appears at about 400 Hz as well, which is far beyond the MESG bandwidth which is typically less than 50 Hz. Note that the difference between Figure 6 and Equation (12) could be mainly attributed to the rounding error and accumulated error of the rebalance loop during numerical computation via Simulink.

## 3. Experiment Results and Discussion

**Figure 9:**
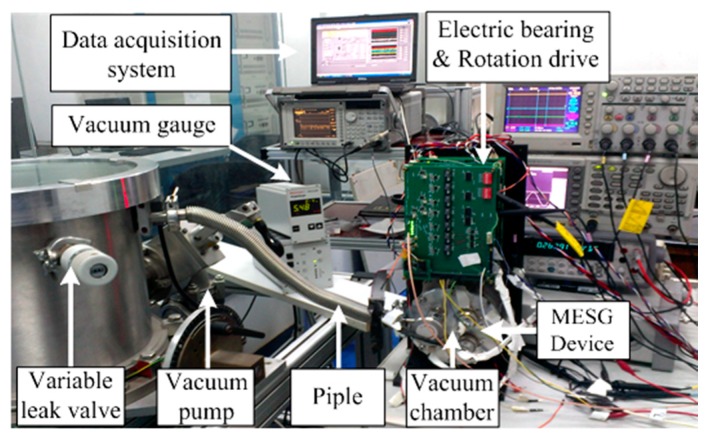
The MESG setup to test the decoupling compensation performance of the gyro rebalance loop.

In our MESG setup, the rebalance loop shares the pick-offs of the angular position and the loop controller with the five-axis suspension loop. A lag-lead compensator is designed to be used as the loop controller G_c_(s), which is realized digitally via a high-speed digital signal processor at a sampling frequency of 20 kHz [21]. The stiffness compensation which eliminates the negative spring effect in the electrostatic torquer acts as a pre-condition for operation of the proposed decoupling compensation realized in the rebalance loop. When the MEMS device runs at the angular rate measurement mode in which the rotor is spinning at a high speed over 10^4^ r/min, the decoupling control compensation (inner-loop and outer-loop decoupling) must operate together to attenuate the inherent cross-axis coupling inside the rebalance loop. Figure 9 illustrates the MESG experiment setup. A small vacuum chamber is used to provide a desirable vacuum condition for the MESG device. It was maintained at high vacuum via a turbo-molecular pump, a vacuum gauge, and associated valves and pipes. The air pressure of the vacuum loop can be adjusted by a variable leak valve. A dynamic signal analyzer (Agilent 35670A) and a data acquisition unit are used in the gyro tests using the proposed stiffness compensation and decoupling control scheme.

### 3.1. Stiffness Compensation

**Figure 10:**
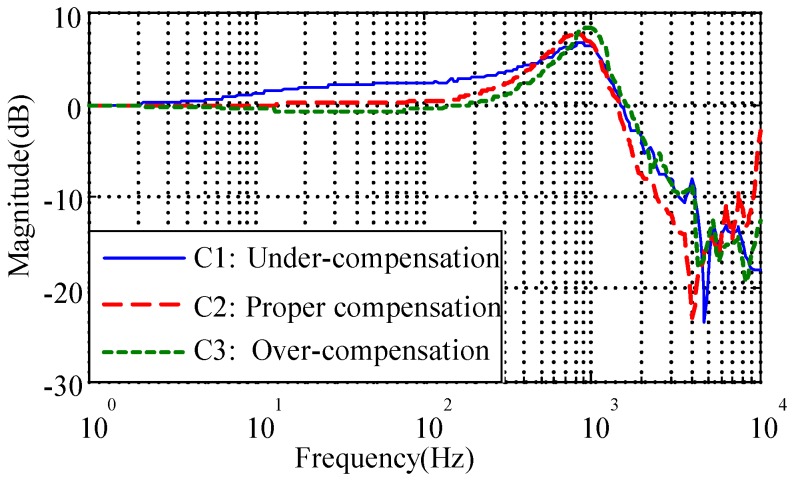
Close-loop frequency responses with different stiffness compensations.

**Figure 11:**
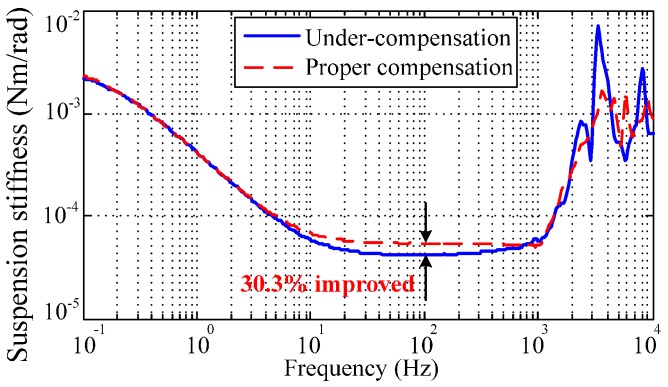
Experimental results of the suspension stiffness, which is improved by 30.3% with proper compensation.

The negative spring effect of the electrostatic torquer has been discussed in Section 2.2. Determining how to verify the accurate stiffness compensation is crucial to obtain desirable full decoupling of the rebalance loop. While the MESG operates in a vacuum greater than 10^−1^ Pa, the control voltages $V_{x},V_{y}$ need to be modified as indicated in Equation (3) to counteract the negative moment from $K_{n}$. Then the compensated results can be observed and evaluated via measuring the closed-loop frequency response and suspension stiffness. To test the closed-loop frequency response, $\varphi_{\mathrm{ex}}(s)$ and $\theta_{\mathrm{ex}}(s)$ are set as the input and output while $\varphi_{\mathrm{ex}}(s)$ and $V_{\mathrm{dx}}(s)$ are set as the input and output in the following suspension stiffness frequency-sweeping test [7]. Three curves C1, C2, and C3 in Figure 10 indicate the under-, proper- and over-compensation in the closed-loop frequency responses, respectively. When the negative stiffness is compensated accurately, such as in C2, the magnitude-frequency curve obviously tends to be flat (at 0 dB) in the middle frequency band (from 10 to 100 Hz), which directly contributes to a stiffness increase of 30.3% compared with the under-compensation of C1, as illustrated in Figure 11. On the other hand, both the under- and over-compensated systems indicate a magnitude deviation from the desirable 0 dB line within the middle frequency range. It is also clear that the proper-compensation system has the smallest resonance peak. The smaller the resonance peak, the more stable the rebalance loop will be. Hence the closed-loop frequency response test is an effective method to observe and evaluate whether the suspension stiffness is compensated properly.

It is noted that the rebalance loop can operate stably without either the stiffness compensation or the decoupling compensation but with degraded performance in this simple operating mode. Moreover, the accurate stiffness compensation is a precondition in order to achieve satisfactory decoupling compensation.

### 3.2. Decoupling Compensation

Considering the MESG is a dual-axis angular rate sensor, when the external angular rate input around one sensing axis occurs, the output signal from the sensing axis corresponding to the input is interested, rather than the output from the crossing axis which is expected to be zero. Nevertheless, the cross-axis coupling between the two sensing axes exists due to the installation misalignment between the MESG’s sensing axis and its holder, imperfect micro-fabrication, and the inherent coupling error of the dual-axis rebalance loop. According to Section 2.4, the verification scheme with an electric angular displacement excitation is one effective way to test the decoupling control regardless of the gyro installation error which dominates in traditional turntable tests.

**Figure 12:**
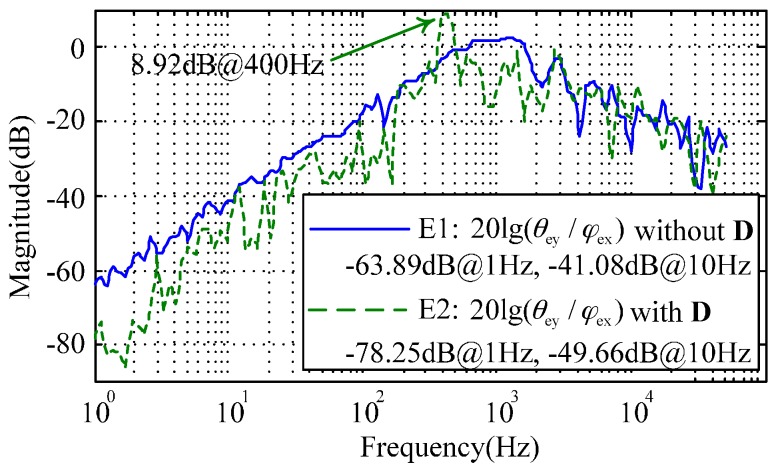
Experimental results with the inner-loop compensation. The angular position coupling is reduced by 14.36 dB at 1 Hz and 8.58 dB at 10 Hz.

When the MESG constantly spins at 1.2 × 10^4^ r/min in a vacuum of 5.5 × 10^−4^ Pa, the inner loop compensator D(s) is introduced into the rebalance loop as shown in Figure 5. By setting $\varphi_{\mathrm{ex}}(s)$ as the input and $\theta_{\mathrm{ey}}(s)$ as the output, the frequency response for the cross-axis angular position coupling was tested by frequency sweeping using 35670A. Figure 12 compares two experimental results with (E2) and without (E1) the inner-loop decoupling compensation. It is clear that the dual-axis coupling of the angular position is attenuated by 14.36 dB at 1 Hz and 8.58 dB at 10 Hz. Besides, the nutation frequency of the MESG is clearly observed and its resonance peak reaches 8.92 dB at 400 Hz. The experimental results roughly agree with the simulation of Figure 6 in the frequency range from 1 to 400 Hz, while the discrepancy may result from the residual air-film damping effect, micro-fabrication tolerances, and the discretization error of the digital controller. It is found that the inner-loop compensator D(s) can effectively reduce the coupling between dual-axis angular position responses, which can be used to verify whether the compensator D(s) operates properly. Note that the input signal $\varphi_{\mathrm{ex}}(s)$ must be limited to less than 10 mV in the decoupling test, or it will result in undesirable saturation of the controller output. On one hand, the input signal and the coupling error are both very small; on the other hand, the micromachining tolerances of the gyro device will result in a small model mismatch. Therefore, the experimental curves E1 and E2 seem noisy.

Further, the outer-loop compensator O(s) is appended into the rebalance loop and aims to reduce the coupling of the two gyroscope output signals. According to the decoupling compensation design in Figure 7 where $\varphi_{\mathrm{ex}}(s)$ is used as the input, the experimental coupling error in $V_{\mathrm{oy}}(s)/\varphi_{ex}(s)$ does not show any obvious improvement when compared to that of $V_{\mathrm{dy}}(s)/\varphi_{ex}(s)$, to which it is similar. It may result from the following limitations. The first limitation is the microfabrication tolerance, especially the mechanical misalignment between the rotor and suspension electrodes, asymmetrical mass misbalance in the axial direction, and the orthogonal error of the two sensing axes. Another limitation is that the second order compensator O(s) is sensitive to the model parameter mismatch of the rebalance loop, which would result in additional errors. Based on the simulation result in Figure 8, the external angular rate input changing in the frequency domain has an obvious impact on the cross-axis coupling of the gyroscope output, rather than the coupling from the installation misalignment which is a fixed value. It seems too difficult to separate the different coupling sources even to measure the cross-axis coupling on a turntable. Therefore, future work towards miniaturizing the MESG prototype and testing on an angular vibration table will be helpful to make a clear verification of the challenging coupling attenuation issue.

## 4. Conclusions

This paper focuses on the analysis and compensation of the cross-axis coupling error which inherently exists in angular motion of the free spinning rotor and limits the accuracy improvement of dual-axis spinning-rotor gyroscopes. Both a stiffness compensation and a decoupling compensation are proposed and investigated to enhance the performance of the MESG rebalance loop. It can be applied to dual-axis spinning-rotor gyroscopes with various suspension schemes, such as liquid-floated, gas-lubricated, electromagnetic and electrostatic bearing, as well as dynamically tuned gyroscopes. The stiffness compensation provides an effective approach to eliminate the negative spring effect of the electrostatic torquer. The proper compensation is not only a prerequisite to ensure the desirable stability of the decoupling control of the rebalance loop, but also contributes to a 30.3% increase of the suspension stiffness by proper compensator design. In addition, we also present an effective scheme to evaluate the effect of stiffness compensation. The rebalance loop model, by considering the residual air-film damping and proper compensation, has been built and shows the magnitude of the inherent coupling error inside the rebalance loop. The proposed dual-loop compensator can attenuate the coupling error by 50.8 dB (from −90.24 dB down to −146 dB) at 1 Hz, which is not easily verified because of the relatively large installation error and small allowable angular-rate frequency range in traditional turntable experiments. Therefore, a test scheme with the electric angular displacement excitation is proposed. It is demonstrated that the inner-loop decoupling method can reduce the cross-axis coupling of the angular position by 14.36 dB at 1 Hz and 8.58 dB at 10 Hz, and also provides an approach to verify whether the decoupling compensation works properly. Future work will focus on further experimental validation of the rebalance loop model, miniaturizing the MESG prototype by MEMS vacuum packaging, and testing the cross-axis coupling on an angular vibration table with higher frequency of the angular rate input. As the gyro coupling error is increased with the excitation frequency, an angular vibration test will be a better solution to investigate the effect of the decoupling compensation and, thus, calibrate the dual-loop compensator parameters.

## Figures and Tables

**Table 1:** Main parameters of the MESG rebalance loop.

Description (unit)	Value
Rotor axial moment of inertia *J_z_* (kg·m^2^)	1.43 × 10^−12^
Rotor radial moment of inertia *J_e_* (kg·m^2^)	7.17 × 10^−13^
Spin rate of rotor ω (rpm)	1.20 × 10^4^
Angular momentum of rotor *H* (kg·m^2^·s^−1^)	1.80 × 10^−9^
Torque-voltage coefficient *K_v_* (Nm·V^−1^)	1.94 × 10^−9^
Voltage amplifier gain *K_a_*	2.54
Angular position stiffness *K_n_* (Nm·rad^−1^)	3.36 × 10^−6^
Sensitivity of position sensor *K_s_* (V·rad^−1^)	5.90 × 10^2^
Stiffness compensation coefficient *K_c_* (V·rad^−1^)	1.16
Squeeze-film damping coefficient *C* (Nm·s·rad^−1^)	4.21 × 10^−11^
Orthogonal-damping elastic coefficient λ (Nm/rad)	5.29 × 10^−8^
Scale factor of the MESG (mV/°/s)	16.05
Measuring range of the MESG (°/s)	±196.1
